# Phase angle and extracellular edema predict risk of postoperative complications in total joint arthroplasties

**DOI:** 10.2478/joeb-2025-0005

**Published:** 2025-03-25

**Authors:** Natalie Nguyen, Michael C. Marinier, Bryan Mouser, Victoria C. Tappa, Marshall Rupe, Jacob M. Elkins

**Affiliations:** University of Iowa, Carver College of Medicine; Department of Orthopedics and Rehabilitation, University of Iowa, Iowa City, IA USA

**Keywords:** Bioimpedance, whole body phase angle, extracellular edema, arthroplasty

## Abstract

**Intro:**

Total knee arthroplasty (TKA) and total hip arthroplasty (THA) are common procedures that improve mobility but carry a risk of postoperative complications, particularly in patients with obesity. Body Mass Index (BMI) is traditionally used for risk assessment but does not account for muscle mass or fat distribution. Bioelectrical impedance analysis (BIA) provides a more detailed body composition evaluation. This study investigates the association between BIA-derived metrics and postoperative complications in TKA and THA, hypothesizing that these metrics are superior predictors compared to BMI.

**Methods:**

A retrospective cohort study was performed on 567 adult patients who underwent primary THA or TKA from January 2020 to December 2023. The data collected included demographic characteristics, comorbidities, preoperative laboratory values, preoperative BIA measurements and postoperative complications. Multivariate logistic regression models were developed to identify independent predictors of postoperative complications. Receiver operating characteristic (ROC) curves assessed the predictive accuracy of BIA-metrics models compared to BMI model.

**Results:**

In a cohort of 567 patients (55.7% female, median age 66), no significant difference in BMI was found between the complication and non-complication groups. However, the complication group had a higher ECW/TBW ratio (0.396 vs. 0.393, p = 0.011), higher ECW/ICW ratio (0.657 vs. 0.647, p = 0.012), and a lower phase angle (4.65 vs. 4.80, p = 0.039). Multivariate logistic regression analysis revealed that higher standardized ECW/TBW (OR 1.65, 95% CI 1.17–2.31, p = 0.004) and ECW/ICW z-scores (OR 1.61, 95% CI 1.15–2.23, p = 0.005) were associated with increased odds of postoperative complications, while a lower phase angle was protective (OR 0.58, 95% CI 0.37–0.91, p = 0.018). ROC analysis showed moderate predictive accuracy for ECW/TBW (AUC 0.71, 95% CI 0.62–0.79), ECW/ICW (AUC 0.70, 95% CI 0.62–0.79), and phase angle (AUC 0.69, 95% CI 0.60–0.79). In contrast, BMI was not significantly associated with complications, and BMI model demonstrated inferior predictive accuracy (AUC 0.61)

**Conclusion:**

ECW/TBW, ECW/ICW and phase angle are associated with postoperative complications in patients undergoing primary TKA or THA. These metrics provide better predictive accuracy than BMI enhancing preoperative risk stratification.

## Introduction

Total knee arthroplasty (TKA) and total hip arthroplasty (THA) are among the most common surgical procedures in the United States, with over 1.4 million cases performed in 2023 alone—a number that continues to rise with an aging population (2). These procedures help patients significantly improve functional status. However, postoperative complications, including periprosthetic fractures, dislocations, infections, and the need for subsequent revision surgery, remain problematic and costly, contributing to over $4 billion USD in annual healthcare expenditures (3).

Obesity is a recognized risk factor for postoperative complications in total joint arthroplasty (TJA). However, due to its role in accelerating osteoarthritis in weight-bearing joints, over 50% of these procedures are performed on obese patients (4,5,6). Obesity has been classified using body mass Index (BMI), defined as weight (kg) divided by height squared (m²). Patients with a BMI of 40 (kg/m^2^) have often been denied surgery due to the increased rates of postoperative complications (7,8,9,10).

However, BMI has increasingly proven to be a suboptimal measure of obesity. It does not account for critical factors such as age, sex, and ethnicity, nor does it provide insight into body fat distribution or the heterogeneity of obesity (11,12,13). Although BMI remains a primary tool for risk stratification in total joint arthroplasties (TJAs) (14,15,16,17,18,19), its effectiveness in predicting outcomes has shown mixed results (20,21,22,23).

Alternative pre-operative risk evaluations have been explored, with body composition measures via bioimpedance analysis (BIA) showing promise (24,25,26,27). Research has shown that body composition metrics, such as percent body fat, and subcutaneous fat thickness, are more predictive of perioperative risks and functional outcomes after TJAs than BMI (24,25,26). Subcutaneous fat thickness has been associated with early re-operation, and infection in morbidly obese patients (25). Additionally, whole-body phase angle, a measure of cellular integrity, has emerged as a potential preoperative marker, correlating with lean tissue measures and age (27). These findings suggest that incorporating body composition assessments can help improve risk stratification and outcomes in total joint arthroplasty.

This study aims to investigate multiple BIA-derived metrics and their associations with postoperative complications following THA and TKA. We hypothesize that BIA-derived metrics are more accurate predictors of postoperative complications than BMI following THA or TKA.

## Materials and Methods

### Study Design

**Figure 1:**
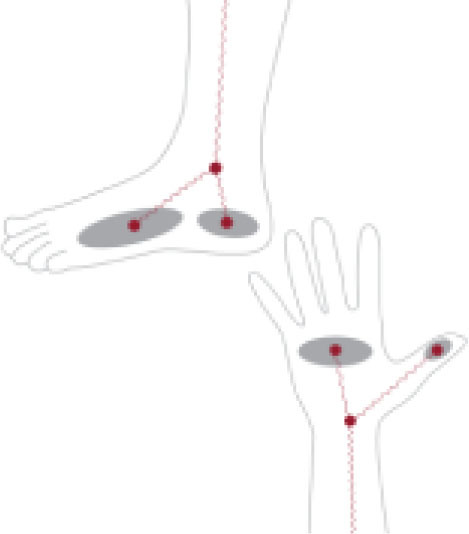
Electrode placement (1).

This study included adult patients (≥ 18 years) who underwent primary TJA from January 1, 2020, to December 31, 2023. Inclusion criteria comprised the following: (1) underwent TKA or THA (2) underwent pre-operative multi-frequency BIA, and (3) had at least one standard-of-care post-operative visit available for evaluation of arthroplasty complication. All BIA measurements were performed as part of standard preoperative assessments within one month of surgery. BIA was conducted using the InBody 770 or 970 (InBody USA, Cerritos, CA, USA) measures impedance at six frequencies (1, 5, 50, 250, 500, and 1000 kHz) and reactance at three frequencies (5, 50, and 250 kHz) which provide a comprehensive assessment of both extracellular and intracellular fluid compartments. Lower frequencies primarily measure extracellular water, whereas higher frequencies assess intracellular water and total body water - this approach is consistent with prior studies evaluating fluid distribution and cellular health using BIA (28, 29). The examination utilized a tetrapolar, eight-point electrode system, with patients standing while electrodes made contact at the feet and hands as shown in **Figure 1** - this system was chosen for its demonstrated reliability in whole-body BIA measurements and minimal operator variability. Patients who were unable to stand for approximately 60 seconds or had an implanted electronic cardiac device were excluded from the study.

### Data Collection

Clinical data and BIA measurements were obtained from 567 patients (311 TKA and 256 THA). All patients underwent only one TJA (either knee or hip) during the study period. Demographics including age, sex, race and comorbidities including diabetes mellitus (DM), hypertension (HTN), immunosuppression, current smoker status, lower extremity lymphedema or edema were collected. Pre-operative laboratory results including hemoglobin A1c (HgbA1c), hemoglobin (Hgb), albumin, and total protein were recorded. Primary TJA complications were documented including deep joint infection, delayed surgical wound healing, dislocation, hardware loosening, revision surgery and emergency department visits related to other postoperative complications. Postoperative data were collected during standard follow-up visits up to 2 years after surgery. This timing was selected to capture early complications while maintaining consistency across the cohort.

BIA parameters reviewed included 50 kHz whole-body phase angle (ϕ), extracellular water : intracellular water (ECW/ICW), extracellular water : total body water (ECW/TBW), percent body fat (PBF), skeletal muscle index (SMI), visceral fat area (VFA) and BMI.

### Statistical Analysis

Patients were divided into non-complication and complication groups based on TJA outcomes. Categorical variables were compared using the Chi-square test or Fisher’s exact test. The Shapiro-Wilk test was used to assess normality. Continuous variables with normal distributions were presented as mean (SD) and compared using the Student t-test, while non-normally distributed variables were presented as median [IQR] and compared using the Mann-Whitney U test. To adjust for multiple comparisons, P-values were corrected using the Benjamini-Hochberg procedure, controlling the false discovery rate at 5%. Multivariate logistic regression analysis was performed to identify independent predictors of postoperative complications. Variables included in the initial models were age, sex, type of TJA (THA or TKA), DM, HTN, immunocompromised status, edema, lymphedema and BIA-metrics. Standardization using z-scores was applied to ECW/TBW and ECW/ICW to facilitate comparison and interpretation. Collinearity among the variables was assessed using the Variance Inflation Factor (VIF), with a VIF ≤ 2 considered acceptable. This step ensured that multicollinearity did not bias the model estimates or inflate standard errors. An iterative backward elimination method was applied, systematically removing variables with P > 0.10 that were consistently non-significant across all models. Model fit was assessed using the Hosmer-Lemeshow test and pseudo-R^2^.The area under the receiver operating characteristic (ROC) curve (AUC) was computed for each multivariate analysis model to evaluate their predictive accuracy in comparison to BMI.

Statistical analyses were performed using SPSS 29.0 (IBM, Chicago, IL), with P < 0.05 considered statistically significant.

### Ethical Approval

Our Institutional Review Board approved this retrospective study (IRB #201904825). A waiver of consent was approved for all subjects. Furthermore, the research related to human use has been complied with all relevant national regulations, institutional policies and in accordance with the tenets of the Helsinki Declaration and has been approved by the authors’ institutional review board or equivalent committee.

## Results

The cohort includes 567 patients, of whom 316 were females (55.7%). The median age was 66 years. Patient demographics and comorbidities are included in **Table 1**. There were no significant differences between the complication and non-complication groups regarding age, sex, race, type of TJA (TKA vs. THA), DM, HTN, smoking status, or lower extremity edema. However, the complication group had a higher proportion of immunocompromised patients (19.0% vs. 10.1%) and individuals with lower extremity lymphedema (4.8% vs. 1.3%), though these differences did not reach statistical significance.

**Table 1:** PATIENT CHARACTERISTICS.

		**TJA complications**		
**Variables**	**Total (n=567)**	**Non-complication (n=525)**	**Complication (n=42)**	**P-value**
Age, median (IQR)	66 [58–72]	67 [59–73]	65 [58–71]	0.588
Sex, female	316 (55.7)	293 (55.8)	23 (54.8)	0.951
TKA	311 (54.9)	286 (54.5)	25 (59.5)	0.527
Race				0.165
White	529 (93.3)	489 (93.1)	40 (95.2)	
Black	21 (3.7)	21 (4.0)	0 (0.0)	
Hispanic	8 (1.41)	7 (1.3)	1 (2.4)	
Asian	6 (1.06)	6 (1.1)	0 (0.0)	
Native Hawaiian/Pacific Islander	2 (0.35)	1 (0.2)	1 (2.4)	
Declined to answer	1 (0.18)	1 (0.2)	0 (0.0)	
Diabetes	103 (18.2)	98 (18.7)	5 (11.9)	0.272
HTN	319 (56.3)	297 (56.6)	22 (52.4)	0.598
Current smoker	9 (1.59)	8 (1.5)	1 (2.4)	0.669
Immunocompromised	61 (10.8)	53 (10.1)	8 (19.0)	0.072
Chronic kidney disease				0.977
Stage 1	3 (0.53)	3 (0.6)	0 (0.0)	
Stage 2	11 (1.94)	10 (1.9)	1 (2.4)	
Stage 3	33 (5.82)	31 (5.9)	2 (4.8)	
Stage 4	4 (0.71)	4 (0.8)	0 (0.0)	
Stage 5	1 (0.18)	1 (0.2)	0 (0.0)	
Lower extremities lymphedema	9 (1.59)	7 (1.3)	2 (4.8)	0.087
Lower extremities edema	48 (8.47)	45 (8.6)	3 (7.1)	0.749

Data are presented as n (%) unless otherwise indicated

Preoperative laboratory data are shown in **Table 2**. No significant differences were observed in hemoglobin A1c, hemoglobin, albumin or total protein levels between the two groups.

**Table 2:** PREOPERATIVE LABORATORY.

**Variables**	**Non-complication (n=525)**	**Complication (n=42)**	**P-value**
HgbA1c	6.53 (0.65)	6.74 (0.74)	0.499
Hgb	13.77 (1.40)	13.52 (1.52)	0.282
Albumin	4.40 [4.20–4.60]	4.40 [4.10–4.50]	0.110
Total protein	7.10 [6.80–7.40]	7.00 [6.80–7.30]	0.929

Data are presented as mean (SD) or median [IQR]

BIA measurements are summarized in **Table 3**. ECW/ICW and ECW/TBW ratios were significantly higher in the complication group and remained significant after FDR adjustment. Although phase angle was initially significant, it did not remain significant after adjustment. No differences were observed for other BIA metrics or BMI.

**Table 3:** UNIVARIATE ANALYSIS.

**Variables**	**Non-complication (n=525)**	**Complication (n=42)**	**P-value**	**Adjusted P-value (FDR)**
ECW/ICW	0.647 [0.632–0.666]	0.657 [0.644–0.673]	0.012*	0.042*
ECW/TBW	0.393 [0.387–0.400]	0.396 [0.391–0.403]	0.011*	0.042*
SMI	8.30 [7.20–9.30]	8.40 [6.80–9.55]	0.651	0.750
PBF	39.50 [32.90–46.80]	38.50 [31.30–46.60]	0.506	0.708
VFA	190 [137–238]	194 [129–234]	0.750	0.750
Phase angle**	4.80 [4.30–5.40]	4.65 [4.13–5.00]	0.039*	0.091
BMI	32.60 [28.30–37.70]	32.00 [26.20–37.70]	0.474	0.708

* Significant correlations are less than 0.05

** 50 kHz – Whole Body Phase Angle

Data are presented as median [IQR]

Four multivariate logistic regression models were developed after multicollinearity testing and iterative backward elimination (**Table 4**). Standardized ECW/TBW and ECW/ICW z-scores were significantly associated with increased complications, with each standard deviation increase raising the odds by 65% and 61%, respectively. Lower whole-body ϕ was associated with higher complication risk, with each unit decrease increasing the odds by 72%. PBF demonstrated a small protective effect but was not consistently significant across models. BMI was not significantly associated with complications.

**Table 4:** MULTIVARIATE ANALYSIS.

**Variables**	**Odds ratio (95% CI)**	**P-value**
**Model 1: ECW/TBW**		
ECW/TBW z-score	1.65 (1.17–2.31)	0.004*
PBF	0.96 (0.93–1.00)	0.048*
Immunocompromised	2.22 (0.95–5.20)	0.065
Albumin	0.48 (0.15–1.60)	0.233

**Model 2: ECW/ICW**		
ECW/ICW z-score	1.61 (1.15–2.23)	0.005*
PBF	0.97 (0.93–1.00)	0.051
Immunocompromised	2.23 (0.95–5.21)	0.064
Albumin	0.47 (0.14–1.57)	0.221

**Model 3: Phase Angle**		
Whole body ϕ	0.58 (0.37–0.91)	0.018*
PBF	0.96 (0.93–1.00)	0.045*
Immunocompromised	2.16 (0.93–5.06)	0.075
Albumin	0.43 (0.13–1.42)	0.167

**Model 4: BMI**		
BMI	1.65 (0.91–1.01)	0.200
Immunocompromised	2.31 (0.98–5.47)	0.063
Albumin	0.49 (0.15–1.67)	0.067

ROC analysis from each multivariate analysis model revealed that all BIA-metric models demonstrated moderate predictive accuracy (AUC range, 0.69–0.71), outperforming the BMI model (AUC = 0.61) (**Table 5**).

**Table 5:** ROC ANALYSIS.

**Models**	**AUC (95% CI)**	**P-value**
Model 1: ECW/TBW	0.71 (0.62–0.79)	<0.001
Model 2: ECW/ICW	0.70 (0.62–0.79)	<0.001
Model 3: Phase Angle	0.69 (0.60–0.79)	<0.001
Model 4: BMI	0.61 (0.51–0.71)	0.032

**Table 6** summarizes postoperative complications observed in 42 patients, including deep joint infections (n=16), delayed wound healing (n=4), dislocations (n=8), arthrofibrosis requiring manual manipulation (n=4), revision surgeries (n=17), and emergency department visits related to surgery (n=4).

**Table 6:** POSTOPERATIVE COMPLICATIONS.

**Type**	**Total (n=42)**	**TKA (n=25)**	**THA (n=17)**
Deep joint infection	16	10	6
Delayed surgical wound healing	4	2	2
Dislocation	8	0	8
Arthrofibrosis*	4	4	0
Revision surgery	17	9	8
ED visits**	4	2	2

* Arthrofibrosis that required manual joint manipulation

** ED visits related to other postoperative complications not accounted for in the above categories.

Data are presented as n

Deep joint infections were more common in TKA cases, whereas dislocations occurred exclusively in THA cases. Arthrofibrosis requiring manual manipulation was observed only in TKA cases. Most ED visits were due to surgical site infections, delayed wound healing, and dislocations, which are reported separately. Therefore, additional ED visits unaccounted for in the above categories were included, specifically for surgical knee pain with suspected extensor mechanism rupture, superficial vein thrombosis at the surgical site, persistent surgical hip pain, and periprosthetic hip fracture.

## Discussion

This study demonstrated that BIA-derived metrics—ECW/TBW, ECW/ICW, and phase angle—are associated with postoperative complications following TJA, outperforming BMI in predictive accuracy. These findings underscore the limitations of BMI as a sole risk stratification tool and highlight the clinical utility of body composition analysis in preoperative assessments.

A higher ECW/TBW ratio is associated with a greater risk of complications post-TJA due to its connection with factors that hinder recovery. These factors include malnutrition, inflammation, and cellular dysfunction (30,31,32,33) – all of which can impair wound healing and increase the risk for postoperative infection (34). Elevated ECW/TBW can reflect fluid imbalances or edema which complicate the post-operative recovery period by exacerbating tissue stress and delaying recovery. Studies have highlighted the role of fluid overload in increasing the risk of surgical site infections, prolonged wound drainage, and the need for re-intervention (30, 31). Therefore, monitoring and managing fluid balance preoperatively and post-operatively is crucial in minimizing complications and improving recovery outcomes.

Similar to ECW/TBW, ECW/ICW also reflects water distribution and can serve as an indicator of hydration status and fluid imbalances. However, its more clinically relevant role lies in its association with muscle strength and quality. Studies have shown that ECW/ICW ratio increases with age and is negatively correlated with muscle strength and physical performance (35, 36). Elevated ECW/ICW has been linked to a higher risk of sarcopenia, particularly in older adults and in patients with obesity or diabetes (37). Sarcopenia, in turn, has been associated with higher rates of post-TJA complications including impaired wound healing, longer hospital stays, and increased susceptibility to infections due to reduced physiological reserves and immune function (38,39,40,41). Patients with sarcopenic obesity face compounded risks because the excess fat mass exacerbates inflammation and metabolic dysfunction, further hindering recovery (42,43,44). Given these associations, ECW/ICW may provide a more nuanced assessment of surgical risk and serve as a complementary metric to BMI for preoperative risk stratification, especially in patients with higher BMI.

A lower whole-body ϕ observed in the complication group suggests compromised cellular integrity. Patients with lower phase angles may have reduced physiological reserves and impaired immune responses, making them more susceptible to complications such as infections, delayed wound healing, and prolonged recovery times. Specifically, lower phase angle values are linked to increased postoperative complications, longer hospital stays, and higher mortality rates, particularly in cardiac and gastrointestinal surgeries (45,46,47). In orthopedic surgery, phase angle can indicate the overall health and nutritional status of the patient (48). Low phase angle correlates with worse functional outcomes after hip fracture rehabilitation (49) and shows potential as a pre-operative risk-marker in TJA (27).

The study did not find significant differences in the prevalence of common co-morbidities like DM and HTN between the groups. However, a higher proportion of immunocompromised patients and those with lower extremity lymphedema were observed in the complication group. Extracellular edema, characterized by high ECW/TBW, and low phase angle, have demonstrated strong diagnostic potential in detecting early lymphedema (50,51,52,53). Early detection and intervention are critical, as persistent edema can delay healing and increase the risk of complications. Evidently, studies have shown that patients with lymphedema undergoing TJA have higher rates of infection, dislocation, reoperation, and revision compared to matched controls (54,55,56). Immunocompromised patients, including those on chronic corticosteroid therapy or with underlying malignancies, may have an increased susceptibility to infections and impaired healing, necessitating more vigilant perioperative management. These conditions may exacerbate the inflammatory response and impede normal wound healing processes, contributing to worse postoperative outcomes.

A key strength of this study is the comprehensive analysis of multiple BIA-derived metrics in a relatively large cohort, enhancing the reliability of our findings. However, the moderate predictive accuracy of AUC indicates that BIA metrics alone may not fully capture the complexity of postoperative risks. Additionally, this is a retrospective, single-center study, which may limit the generalizability of the results. Residual confounders, such as physical activity levels and nutritional status, were not measured and could influence outcomes.

Despite the moderate predictive accuracy, the superior performance of BIA metrics over BMI suggests significant clinical value, supporting their integration into preoperative risk assessments. Future studies should aim to develop composite risk scores or stratification tools incorporating BIA metrics alongside traditional clinical variables like BMI, immunosuppression, smoking status, and uncontrolled diabetes mellitus. Leveraging the predictive power of ECW/TBW, ECW/ICW, and phase angle—metrics sensitive to fluid imbalances, cellular health, and nutritional status—could enable a more personalized risk assessment strategy for TJA patients. This approach would enhance perioperative care by accounting for complex interactions between body composition and clinical variables.

Future studies should also consider incorporating larger patient populations and multi-center data to enhance the generalizability of the findings. This approach would validate the observed associations across diverse healthcare settings, ensuring broader applicability and more robust clinical guidelines.

In conclusion, this study highlights the potential of BIA-derived metrics, especially the ECW/TBW and phase angle, as predictors of postoperative complications in THA and TKA. The results emphasize the need for a more comprehensive preoperative assessment that includes detailed body composition analysis in addition to the traditional reliance on BMI alone.
